# The Effectiveness and Usefulness of Assistive Technology Training in Building Workforce Capacity for Rehabilitation and Healthcare Professionals in the MENA Region: A Mixed-Methods Study

**DOI:** 10.3390/healthcare14101362

**Published:** 2026-05-15

**Authors:** Hassan Izzeddin Sarsak

**Affiliations:** Occupational Therapy Program, Batterjee Medical College, Jeddah 21442, Saudi Arabia; hassan.sarsak@bmc.edu.sa

**Keywords:** assistive technology, capacity building, healthcare and rehabilitation, MENA region, universal health coverage, workforce development

## Abstract

**Highlights:**

**What are the Implications of the Main Findings?**

- **Workforce Competency and Efficiency:** Providing healthcare professionals with standardized assistive technology training, such as the WHO-TAP and ISWP-based modules, directly addresses the lack of a trained workforce, ensuring that services are delivered by competent personnel.
- **Enhanced Patient Outcomes:** By equipping primary healthcare personnel with the skills to provide appropriate assistive products, the program facilitates the functional independence and social inclusion of persons with disabilities of all ages.
- **Support for Universal Health Coverage (UHC):** Integrating assistive technology training into the broader workforce helps countries meet global health targets and Sustainable Development Goal (SDG) #3 (Health and Wellbeing) by increasing regional access to affordable, high-quality assistive technology.
- **Standardization of Clinical Practice:** The use of evidence-based learning and global roadmap initiatives ensures that healthcare and rehabilitation specialists across different countries in the MENA region apply a consistent “5 Ps” model (People, Products, Provision, Personnel, and Policy) and quality assistive technology service provision in their practice.
- **Interdisciplinary Collaboration:** The training highlights the multi-disciplinary nature of the field, encouraging better communication and role-sharing between occupational therapists, physiotherapists, nurses, and other stakeholders.

**Abstract:**

**Purpose:** Access to assistive technology (AT) is a fundamental human right and a critical component of Universal Health Coverage (UHC). In the Middle East and North Africa (MENA) region, the scarcity of trained professionals remains a significant barrier to AT service provision. This study evaluates the effectiveness and perceived usefulness of the Assistive Technology Training Program (ATTP), a specialized continuing education initiative designed to build workforce capacity among rehabilitation and healthcare professionals. **Methods:** A convergent mixed methods design was used to analyze quantitative pre/post-test scores and qualitative focus group open-ended responses. Quantitative data were gathered from 386 participants across 11 MENA countries using a pre- and post-test assessment of AT knowledge. Qualitative utility and participant satisfaction were assessed through a 5-point Likert scale survey evaluating content relevance, trainer expertise, and facilities. Association tests (ANOVA and *t*-tests) were conducted to identify factors influencing knowledge gain. **Results:** Participants demonstrated a statistically significant improvement in AT knowledge, with the overall mean score increasing from 3.67 ± 1.13 to 7.50 ± 1.25 (*p* < 0.001). High levels of satisfaction were reported, with 92% of participants rating the training as “Very Good” or “Excellent” regarding its relevance to clinical needs. Association tests revealed that professional background (*p* < 0.001), employment status (*p* = 0.0017), level of education (*p* = 0.011), and prior training experience (*p* = 0.026) were significant factors in the magnitude of improvement, although all subgroups achieved significant learning gains. Qualitative thematic analysis per the focus group discussions using the WHO-GATE 5 P framework identified three major themes: (1) Structural Challenges: Issues with Products and Provision point toward a need for better infrastructure and localized supply chains. (2) Human Capital: Personnel barriers emphasize that training shouldn’t just be for professionals, but should extend to caregivers as well. (3) Systemic and Social Change: Policy and People focus on the “soft” side of AT moving toward user-involved guidelines and fighting social stigma to ensure rights are upheld. **Conclusions:** The ATTP is an impactful educational intervention that significantly enhances the foundational competencies of healthcare professionals in the MENA region. By addressing knowledge gaps and fostering practical skills, the program serves as a preliminary model that demonstrates potential for building regional capacity and supporting the United Nations’ Sustainable Development Goal (SDG) #3 related to health and wellbeing and SDG #4 related to quality education and lifelong learning opportunities for all. Further research is required to evaluate its long-term scalability and clinical impact.

## 1. Introduction

The global landscape of healthcare is shifting toward a more inclusive model where assistive technology (AT) is recognized not as a luxury, but as a necessity for functional independence and social participation [1]. According to the World Health Organization (WHO), over 2.5 billion people worldwide require one or more assistive products, a number expected to grow to 3.5 billion by 2050 [2]. Despite this need, the “occupational justice” gap remains wide, particularly in low- and middle-income countries where access to AT can be as low as 3% for some populations [3].

In the Middle East and North Africa (MENA) region, the challenges are multifaceted. Socio-political instability, varying levels of economic development, and a lack of standardized educational programs for healthcare and rehabilitation professionals have created a significant deficit in the AT workforce [4,5]. Healthcare and Rehabilitation professionals including occupational therapists, physiotherapists, and nurses often graduate without specialized training in the assessment, fitting, and follow-up processes required for complex assistive devices [2,6]. To bridge this gap, the Assistive Technology Training Program (ATTP) was developed. This program is a specialized continuing education course that integrates global best practices from the WHO Training in Assistive Products (TAP), the International Society of Wheelchair Professionals (ISWP), and the Rehabilitation Engineering and Assistive Technology Society of North America (RESNA). The ATTP adopts the WHO-GATE 5 P framework model (People, Products, Provision, Personnel, and Policy) to provide a comprehensive framework for service delivery [7,8].

While global roadmaps provide foundational frameworks, independent empirical studies in low- and middle-income countries (LMICs) underscore that the primary barrier to assistive technology access is often the misalignment between available technology and the lived realities of users [9]. Research highlights that systemic disadvantages, such as limited spatial autonomy and social exclusion, frequently undermine the effectiveness of assistive technology interventions when not supported by a trained workforce [9,10,11]. Furthermore, recent studies in the MENA region suggest that even when professionals are aware of advanced low-vision aids, high costs and a lack of specific institutional training remain significant deterrents to clinical adoption [12,13,14]. Despite the recognized need for AT workforce development, a significant knowledge gap persists regarding the most effective educational strategies for the MENA region’s unique socio-political and linguistic landscape [2,3]. Most existing studies on AT training are localized to single institutions or specific high-income countries, leaving it unclear whether a standardized, multi-national curriculum can effectively address the regional deficit [2,3,4,5,6]. Furthermore, there is a lack of research that moves beyond simple knowledge acquisition to explore how these learned competencies intersect with systemic regional barriers like supply chain instability and social stigma [6,7,8]. This study addresses these gaps by evaluating the ATTP across 11 MENA countries. It advances the literature not only by measuring knowledge gains in a diverse professional cohort but also by utilizing a mixed-methods approach to identify the specific facilitators and structural challenges that dictate the real-world utility of AT education in this region. Therefore, this study aims to evaluate the impact of the ATTP on a large, multi-national cohort of 386 healthcare and rehabilitation professionals and students from 11 countries in the MENA region. By measuring knowledge acquisition and participant satisfaction, this research seeks to validate a scalable model for regional workforce development that supports the road to universal health coverage.

## 2. Materials and Methods

### 2.1. Study Design

A mixed-methods approach was adopted to capture both the objective learning outcomes (quantitative) and the subjective experience of the participants (qualitative). This design allowed for a robust triangulation of data, ensuring that the results reflect both clinical competency and the practical utility of the curriculum [15,16]. More specifically, this study employed convergent mixed methods design to simultaneously collect and analyze quantitative pre/post-test scores and qualitative open-ended responses. This approach aimed to triangulate learning outcomes with participant-identified barriers and facilitators in the MENA region.

### 2.2. Participants and Setting

**Figure 1:**
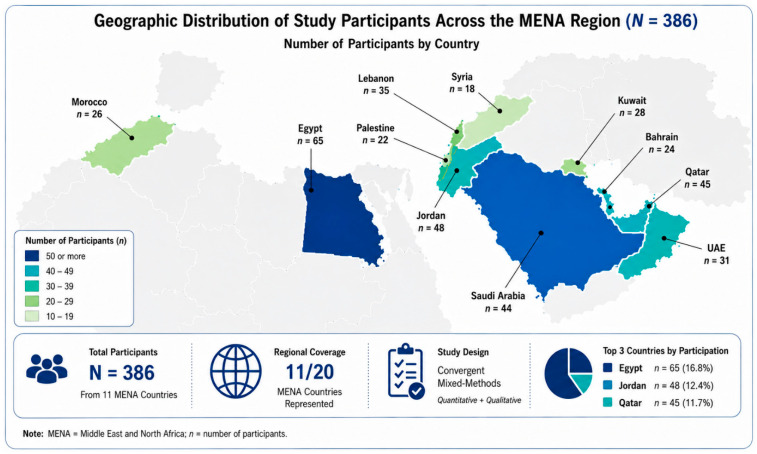
Geographical Distribution of the 11 Participating Countries across the Middle East and North Africa (MENA) Region Included in the Study.

A total of 386 participants were recruited from 11 countries: Bahrain, Egypt, Jordan, Kuwait, Lebanon, Morocco, Palestine, Qatar, Saudi Arabia, Syria, and the United Arab Emirates (see Figure 1). The cohort was diverse, consisting of 205 Occupational Therapists, 92 Physiotherapists, 28 Speech and Language Therapists, 16 Prosthetics and Orthotics specialists, and other allied health professionals. Notably, 337 were undergraduate students or recent graduates, while 49 held postgraduate degrees. The recruitment of participants for the ATTP followed a multi-channel outreach strategy designed to engage a diverse cohort of healthcare professionals across the MENA region. Prospective participants were identified and invited by our multi-channel outreach through professional networks, academic institutions, and clinical partners across the 11 participating countries. Information regarding the training was disseminated via professional mailing lists, social media platforms dedicated to healthcare and rehabilitation sciences, and official announcements at participating medical facilities. Interested individuals registered through an online portal, where they provided demographic and professional background information to ensure a representative sample of the regional workforce.

Participation inclusion criteria were (1) Professional Status: Practicing healthcare and rehabilitation professionals (e.g., Occupational Therapists, Physiotherapists, Speech-Language Pathologists, Nurses, and Prosthetists/Orthotists) or senior-level undergraduate and/or postgraduate students in these disciplines; (2) Geographical Location: Residents or practitioners currently based in one of the 11 targeted MENA countries (Jordan, Lebanon, Saudi Arabia, Egypt, Bahrain, Palestine, Kuwait, UAE, Syria, Morocco, and Qatar); (3) Language Proficiency: Ability to understand and engage with instructional materials delivered in English or Arabic, as the program utilized bilingual clinical frameworks; and (4) Commitment: Willingness to complete both the pre-training and post-training knowledge assessments and the full 8-h intensive curriculum. Exclusion criteria were (1) Non-Related Fields: Individuals working in administrative or non-clinical roles that do not involve direct patient care or the provision of assistive products; (2) Incomplete Participation: Participants who were unable to attend the full duration of the training or failed to complete either the pre-test or post-test assessments; and (3) Duplicate Attendance: Individuals who had previously attended an identical ATTP session were excluded to prevent data redundancy and ensure the assessment of new knowledge gain.

### 2.3. The Assistive Technology Training Program (ATTP)

The ATTP is an intensive, 8-h continuing education course designed to equip rehabilitation and healthcare professionals with the practical skills necessary for appropriate assistive technology (AT) service delivery. The curriculum integrates evidence-based frameworks from the World Health Organization (WHO), the International Society of Wheelchair Professionals (ISWP), the Rehabilitation Engineering and Assistive Technology Society of North America (RESNA), and many other reliable resources in the field of assistive technology service provision [17,18,19,20,21,22,23,24]. The program was led by a WHO consultant and certified wheelchair trainer, focusing on cultural adaptation and occupational justice within the MENA region. Instruction utilized a case-based approach, combining theoretical lectures with hands-on practical sessions in six primary AT streams: cognition, communication, vision, hearing, self-care, and mobility.

The program curriculum included (1) Global Roadmap: Discussion on the role of AT in empowering persons with disabilities (PWD) and global healthcare initiatives, contributions, and future directions towards fostering universal access to assistive technology; (2) the WHO-TAP Integration: Introduction to the WHO Training in Assistive Products (TAP) digital platform and registration process; (3) Practical Skills: Hands-on assessment and fitting for a variety of assistive products categorized into the main AT streams including cognition, communication, vision, hearing, self-care, and mobility assistive technology devices; (4) assistive technology service provision models, the WHO assistive technology service provision-recommended steps, and the assistive technology outcome measures; and (5) Intervention and Compensatory Approaches: Case-based problem-solving therapy (PST) for activities of daily living (ADL) solutions. By the conclusion of the training, participants were expected to: (1) apply the “5 Ps” model (People, Products, Provision, Personnel, and Policy) to facilitate effective, high-quality AT service provision, (2) demonstrate proficiency in selecting, fitting, and assessing products from the WHO Priority Assistive Products List (APL), (3) understand the interdisciplinary nature of the field, including the roles of stakeholders, caregivers, and end-users in improving functional outcomes, and (4) identify regional barriers and collaborative strategies to integrate AT into national healthcare and education systems, supporting the road to Universal Health Coverage (UHC).

### 2.4. Data Collection and Analysis

A 10-point pre-test was administered before the session, followed by an identical post-test at the conclusion (see Supplementary Materials). To ensure the scientific rigor of the quantitative analysis, the 10-item pre- and post-test assessment underwent a formal validation process to establish its psychometric properties. Content validity was established by a panel of three independent experts in assistive technology and rehabilitation who mapped each item to the WHO-TAP core competencies. The assessment utilized a 10-point scale consisting of case-based, multiple-choice questions designed to measure clinical reasoning across six primary AT streams: cognition, communication, vision, hearing, self-care, and mobility. *Example Assessment Item:* “A 65-year-old client with limited shoulder range of motion and grip strength requires an assistive product for independent feeding. Based on the WHO Priority Assistive Products List (APL), which features are most critical to assess when selecting a modified utensil for this user?”. A pilot study (n = 20) yielded a Cronbach’s alpha coefficient of 0.84, indicating high internal consistency. The instrument’s construct validity was further confirmed by its ability to distinguish between participants with varying levels of prior expertise. Similarly, the 5-point Likert scale satisfaction survey (1 = Poor, 5 = Excellent) was validated through an expert panel and a pilot study with 22 professionals to rectify phrasing ambiguities. This instrument demonstrated exceptional stability with a Cronbach’s alpha of 0.94. Items were categorized into four domains: Training Content, Trainer Evaluation, Facilities Evaluation, and Overall Impression.

Data were analyzed using Python 3.14 (pandas, scipy, statsmodels) [25]. The assumptions for parametric testing, including normality (Shapiro–Wilk test) and homogeneity of variance (Levene’s test), were verified prior to analysis. Paired *t*-tests determined the significance of knowledge improvement, with effect sizes calculated using Cohen’s d. ANOVA (Analysis of Variance) was used to test associations between demographic factors and the degree of improvement, with partial eta squared (n^2^) reported to indicate effect size magnitude.

Qualitative data were derived from open-ended survey responses provided by all 386 participants and supplemental focus group discussions. A total of six focus groups were conducted (averaging 8–10 participants per group), with participants purposively selected to ensure representation across the 11 MENA countries and primary professional disciplines (Occupational Therapy, Physiotherapy, and Speech-Language Pathology). To ensure the credibility and reproducibility of the findings, the following coding procedure was implemented: (1) *Coding Framework:* A deductive thematic analysis was conducted, using the five domains of the WHO-GATE 5 P framework (People, Products, Provision, Personnel, and Policy) as the primary coding categories. (2) *Coding Procedure:* Two independent coders, specialists in rehabilitation and assistive technology, initially reviewed the raw transcripts and open-ended responses to identify recurring units of meaning. These units were then categorized into the 5 P domains. (3) *Discrepancy Resolution:* Following the initial coding, the two researchers met to compare their findings. Any discrepancies in code assignment or thematic categorization were resolved through collaborative discussion until 100% consensus was reached. (4) *Expert Verification:* To further ensure rigor, the final thematic map and categorized strategies were reviewed by a third independent expert (an ISWP Certified Wheelchair Trainer and WHO consultant) to confirm that the interpretations remained grounded in the data and aligned with global clinical standards. This rigorous process allowed for the triangulation of objective learning outcomes with the subjective, context-specific challenges identified by healthcare professionals across the 11 participating MENA countries.

### 2.5. Ethical Approval and Participant Consent

This study was approved by the WHO ERC (Master Protocol ERC.0003846) and the research ethical committee at Batterjee Medical College (Proposal Approval RES-2023-0088). All 386 participants provided informed consent prior to their inclusion in the study. The consent process involved a detailed explanation of the study’s purpose, the nature of the pre- and post-test assessments, and the qualitative focus group discussions. Participation was strictly voluntary, and individuals were informed of their right to withdraw from the training or the data collection process at any time without penalty or impact on their professional or academic standing. To ensure participant privacy, all data collected through the online portal and physical assessments were de-identified and assigned unique alphanumeric codes. Raw data were stored on a password-protected server accessible only to the primary investigator to maintain strict confidentiality throughout the analysis and manuscript preparation.

## 3. Results

### 3.1. Participant Demographics

The study successfully engaged a robust and diverse cohort of 386 participants representing 11 countries across the MENA region. The demographic profile reflects a young, emerging workforce, with a mean age of 23.8 years. Gender distribution was predominantly female (81.3%), consistent with global trends in the allied health and rehabilitation professions. In terms of professional background, Occupational Therapy (53.1%) and Physiotherapy (23.8%) constituted the largest groups, followed by Speech and Language Therapy and other specialized rehabilitation disciplines. A significant portion of the sample (87.3%) consisted of undergraduate students or recent graduates, highlighting the program’s role in foundational capacity building. Furthermore, while 65.5% of participants reported some form of previous exposure to assistive technology, the baseline knowledge scores suggested a critical need for the standardized, intensive training provided by the ATTP (see Table 1).

### 3.2. Quantitative Findings: Knowledge Acquisition

The primary measure of effectiveness was the change in test scores. The overall mean pre-test score was 3.67 ± 1.13, while the post-test score significantly improved to 7.50 ± 1.25 (*p* < 0.001). This represents a large effect size (Cohen’s d = 3.21), indicating a substantial practical impact on participant knowledge. Table 2 represents the knowledge improvement scores by country.

### 3.3. Quantitative Findings: Participant Satisfaction and Training Utility

The survey revealed high levels of reported satisfaction across all 11 participating countries, though these findings should be interpreted with the caution warranted by the identified ceiling effect. To better illustrate the response variability, Table 3 includes the frequency distribution for each category. Every participant rated the clarity of objectives, trainer expertise, and overall engagement as “Excellent” (5/5), resulting in a mean score of 5.00 for these items. While a slight distribution was noted in areas such as “On-site assistance” (4.75) and “Time allotment” (4.82), the majority of responses remained in the top two tiers (“Very Good” or “Excellent”). The high concentration of “Excellent” ratings suggests a potential ceiling effect, particularly regarding trainer performance and core curriculum objectives. This may be influenced by the immediate post-training timing of the survey and the specialized nature of the content for this cohort. While the data indicate a positive reception, the high concentration of ‘Excellent’ ratings suggests that the findings primarily reflect the participants’ immediate enthusiasm and perceived relevance of the content rather than a definitive appraisal of long-term educational utility.

### 3.4. Association Tests

Based on the statistical analysis (ANOVA) performed on the dataset of 386 participants, Table 4 summarizes the factors associated with the degree of knowledge improvement, including F-statistics, *p*-values, and partial eta squared (ηp^2^) values. Occupational therapy and physiotherapy students showed the highest rates of improvement compared to general nursing students (*p* < 0.001, ηp^2^ = 0.11). Undergraduate students demonstrated a significantly higher mean gain compared to postgraduate participants (*p* = 0.011, ηp^2^ = 0.06). Factors influencing the improvement in test scores (Improvement = Post-test − Pre-test) were *(1) Profession (p < 0.001):* Occupational therapy and physiotherapy students showed the highest rates of improvement compared to general nursing students, *(2) Employment Status (p = 0.0017):* Currently employed professionals had higher baseline scores but slightly smaller gains compared to students, who started with less knowledge but showed significant rapid acquisition, *(3) Education Level (p = 0.011):* Undergraduate students demonstrated a significantly higher mean gain compared to postgraduate participants, and *(4) Previous Training (p = 0.026):* Participants who had never received prior AT training experienced the most substantial improvement, confirming the program’s utility for entry-level capacity building.

### 3.5. Qualitative Findings: Thematic Analysis of Barriers and Facilitators

Per the thematic analysis of focus group discussions and trainee responses, the following supported themes emerged:

#### 3.5.1. Theme 1: Structural Challenges (Products and Provision)

Participants identified significant barriers regarding the availability and localized supply of assistive products. Representative Quotation: *“We often know what the patient needs after the training, but the high cost and lack of local suppliers mean the right product never reaches the clinic.”*

#### 3.5.2. Theme 2: Human Capital (Personnel)

Personnel barriers emphasize that competency must extend beyond the professional to include the immediate support system. Representative Quotation: *“Training for us [professionals] is the first step, but without involving the caregivers and families in the process, the device often ends up unused in a corner.”*

#### 3.5.3. Theme 3: Systemic and Social Change (Policy and People)

This focuses on the “soft” side of AT, such as user-involved guidelines and the fight against social stigma. Representative Quotation: *“Even when we provide the technology, social stigma remains a huge wall. We need national policies that promote social acceptance and treat AT as a fundamental human right, not a charity.”*

Using the 5 Ps model, participants proposed strategies for improvement related to: *Products*, enhancing quality through standardized product standards and exploring local manufacturing; *Provision*, expanding referral systems and integrating AT into health, education, and social welfare ministries; *Personnel*, prioritizing pre-service training and the involvement of users and caregivers in the delivery process; *Policy*, establishing consistent financing mechanisms and national monitoring systems; and *People*, advocating for AT as a fundamental human right and promoting social acceptance (see Table 5).

## 4. Discussion

The results of this study indicate a significant association between the ATTP intervention and the acquisition of foundational assistive technology knowledge. Consistent improvement across diverse regional cohorts suggests a widespread knowledge gap and underscores the program’s potential as a regional capacity-building tool. However, while these initial results are encouraging, the long-term scalability of the ATTP model requires further validation through longitudinal study and more diverse recruitment strategies to account for potential selection bias.

Summary of key findings from association tests in the current study confirms results from previous studies. Occupational Therapy and Physiotherapy cohorts showed distinct patterns of improvement compared to general healthcare disciplines, likely due to the highly relevant nature of the assistive technology modules to their core scope of practice [26,27]. Undergraduate students demonstrated significantly higher mean gains compared to postgraduate or employed professionals. Interestingly, those with no prior training showed the most substantial leap in scores (*p* = 0.0266). This confirms the program’s effectiveness in taking participants from a low baseline to a high level of competency in a single intensive session. The strong favorable response reported by participants suggests an appreciation for the ATTP curriculum in a region where such specialized training is scarce. However, this near-unanimous feedback likely reflects a ‘honeymoon effect’ common in short-term intensive workshops, rather than a nuanced evaluation of the pedagogical structure. The high concentration of “Excellent” ratings frequently reaching 5.00 suggests a potential ceiling effect, particularly regarding trainer performance and the clarity of core curriculum objectives. This suggests that while the program met or exceeded expectations, the current Likert-scale instrument may lack the sensitivity to distinguish between high levels of satisfaction and exceptional performance. This phenomenon may be partially attributed to the specialized nature of the content; for a cohort predominantly composed of undergraduate students with limited prior exposure, the intensive and hands-on nature of the ATTP may have appeared uniquely impactful compared to their standard academic curricula. The heavy weighting of the sample toward undergraduate students (87.3%) and recent graduates highlights the ATTP’s role as a foundational intervention. However, the implications for workforce development differ significantly across career stages. For students, the program bridges the ‘training-practice gap’ by providing standardized skills often missing from traditional academic curricula. In contrast, for the 12.7% of postgraduate and experienced participants, the lower rate of knowledge gain (*p* = 0.011) may suggest a ‘ceiling effect’ due to higher baseline scores or a need for more advanced, specialized training modules rather than foundational ones. While early-career training is essential for long-term regional capacity, addressing the needs of the existing senior workforce requires a nuanced approach that accounts for their established clinical workflows and the specific systemic barriers they encounter in leadership or specialized roles.

### 4.1. Capacity Building and Global Impact

The ATTP aligns with the WHO’s initiative to prepare a broader workforce to fulfill an assistive technology role. By focusing on the “5 Ps” model, the training moves beyond mere product selection and emphasizes the socio-political and systemic factors that influence assistive technology access [7,8]. The substantial leap in scores among participants with no prior training (*p* = 0.0266) highlights the ATTP’s potency as a foundational capacity-building tool. Integrating this standardized curriculum into early-career academic programs could bridge the ‘training-practice gap,’ ensuring that the emerging workforce is ‘AT-ready’ upon graduation [28,29,30,31,32,33,34]. The integration of qualitative data provides a deeper understanding of why knowledge acquisition alone is insufficient for regional change. For example, while participants gained significant knowledge, their identification of “supply chain challenges” and “poor availability of appropriate training” suggests that clinical competency must be supported by policy-level interventions. The proposed strategies, such as strengthening product standards and fostering multi-disciplinary collaboration, align with the need for a holistic approach to meeting Sustainable Development Goal #3. Furthermore, the emphasis on user-involvement highlights a shift toward more person-centered care in the MENA region [35].

### 4.2. Implications for Healthcare and Rehabilitation

The findings of the current study suggest that the ATTP has the potential to support the advancement of healthcare systems in the MENA region by addressing critical workforce knowledge gaps. By demonstrating significant knowledge gain across 11 countries, the program offers a preliminary model that aligns with the global mandates of Sustainable Development Goal (SDG) 3 and SDG 4 [36]. However, it is important to note that while expanding technical capacity is a prerequisite for achieving Universal Health Coverage (UHC), this study does not measure system-level transformation [37]. The transition from individual knowledge acquisition to enhanced patient outcomes and functional independence for persons with disabilities depends on broader systemic factors, such as policy support and supply chain stability, which were identified by participants but not directly influenced by this educational intervention [38,39,40]. The significant improvement in knowledge scores among diverse healthcare and rehabilitation professional groups in this study aligns with emerging competency frameworks that advocate for interdisciplinary assistive technology implementation. Independent research suggests that integrating knowledge from physical therapy, occupational therapy, and speech-language pathology is critical for maximizing functional outcomes, particularly for populations with complex motor and cognitive needs. By fostering a community of practice, interdisciplinary training models help bridge the gap between theoretical knowledge and the practical assessment, provisioning, and follow-up steps required in the assistive technology service delivery pathway [41,42,43]. The identification of social stigma as a barrier in this study is corroborated by socio-technical research, which argues that technical opportunities can only reach their full potential when they address adverse social contexts [9]. Empirical evidence from other LMIC settings indicates that abandonment rates remain high when cultural dimensions and emotional experiences are overlooked in the training and prescription process. Consequently, training must go beyond device-specific instruction to include strategies for promoting social acceptance and user rights [44].

### 4.3. Limitations and Future Directions

The primary limitation of this study is its single-group pre–post design without a control or comparison group. This design choice limits the ability to definitively attribute knowledge gains exclusively to the ATTP, as alternative factors, such as testing effects (familiarity with the assessment items) or short-term recall, cannot be fully excluded. Additionally, while these findings demonstrate a significant short-term educational impact, they do not reflect long-term knowledge retention or the direct translation of competencies into clinical practice. As knowledge acquisition is a prerequisite for, rather than a sole predictor of, clinical behavior change, these results should be viewed as preliminary evidence of educational efficacy rather than sustained professional mastery. Future research should employ longitudinal designs to evaluate the durability of these learning gains over time. Furthermore, follow-up studies are essential to assess the impact of training translating into tangible changes in clinical practice or improved functional outcomes, such as the quality of assistive technology service provision and the long-term functional independence of the patients served by the participants. Also, the use of identical pre- and post-test instruments may have introduced a potential recall bias, which could contribute to the inflation of post-test scores. While the technical complexity of the case-based questions was intended to minimize this effect, future studies should consider utilizing randomized item banks or alternate forms of assessment to further mitigate testing effects. Future research should also evaluate the long-term clinical outcomes of the participants’ patients and the sustainability of the skills learned. Furthermore, the dual role of the primary investigator as the developer and lead trainer of the ATTP presents a potential risk of bias. While this was disclosed, it may have influenced participant responses through social desirability bias. Consequently, the high satisfaction and utility ratings should be viewed as an indicator of participant engagement and subjective approval rather than an objective measure of the program’s superiority over other potential educational models. Participants might have felt inclined to provide favorable feedback to the instructor. To mitigate this risk, the study utilized a validated, objective pre- and post-test assessment to measure knowledge acquisition, which demonstrated statistically significant gains independent of subjective satisfaction. Future iterations of the study could utilize independent, third-party evaluators for both training delivery and data collection to further enhance the objectivity of the findings. Additionally, the demographic composition, predominantly of undergraduate students, limits the generalizability of these results to the wider population of experienced, long-term healthcare practitioners in the MENA region. While the program was effective for entry-level capacity building, the findings cannot be extrapolated to predict how highly experienced professionals might respond to the same curriculum. Future studies should aim for a more balanced recruitment of mid-career and senior professionals to evaluate the program’s utility as a re-certification or advanced professional development tool.

## 5. Conclusions

The ATTP serves as a promising preliminary model for foundational workforce development, yielding substantial immediate knowledge gains and high levels of reported trainee approval across the MENA region. However, given the single-group design and the focus on immediate assessment, these findings represent preliminary educational impact. Future longitudinal research and randomized controlled trials are essential to evaluate the durability of these gains and their actual translation into improved service quality and patient outcomes. Moving forward, longitudinal research and randomized controlled trials are essential to evaluate the durability of these learning gains and their actual translation into improved service quality. Integrating such evidence-based models into national healthcare strategies remains a target for supporting Universal Health Coverage, provided their long-term efficacy is established through further study.

## Figures and Tables

**Table 1:** Demographic Characteristics of Participants (N = 386).

Characteristic	Category	Frequency (n)	Percentage (%)
**Gender**	Female	314	81.3%
	Male	72	18.7%
**Country**	Egypt	65	16.8%
	Jordan	48	12.4%
	Qatar	45	11.7%
	Saudi Arabia	44	11.4%
	Lebanon	35	9.1%
	UAE	31	8.0%
	Kuwait	28	7.3%
	Morocco	26	6.7%
	Bahrain	24	6.2%
	Palestine	22	5.7%
	Syria	18	4.7%
**Profession**	Occupational Therapy	205	53.1%
	Physiotherapy	92	23.8%
	Speech & Language Therapy	28	7.3%
	Other	61	15.8%
**Education Level**	Undergraduate	337	87.3%
	Postgraduate	49	12.7%
**Employment**	Employed	195	50.5%
	Student	191	49.5%
**Prior AT Training**	Yes	253	65.5%
	No	133	34.5%

Other (Prosthetics and Orthotics, Physical Medicine and Rehabilitation, Nursing, Rehab Engineering, Special Education).

**Table 2:** Knowledge Improvement Scores by Country (N = 386).

Country	Participants (n)	Pre-Test Mean (SD)	Post-Test Mean (SD)	Mean Gain	t-Statistic	*p*-Value
**Palestine**	22	3.09 (0.89)	7.61 (1.17)	4.52	19.34	<0.001
**Syria**	18	3.53 (0.87)	8.06 (0.78)	4.53	16.52	<0.001
**Qatar**	45	3.67 (1.12)	8.09 (1.11)	4.42	23.11	<0.001
**Kuwait**	28	3.30 (0.84)	7.71 (1.08)	4.41	18.25	<0.001
**UAE**	31	3.56 (1.08)	7.94 (1.12)	4.38	20.44	<0.001
**Jordan**	48	2.69 (0.95)	6.84 (1.47)	4.15	24.67	<0.001
**Lebanon**	35	3.84 (1.16)	7.29 (1.51)	3.45	14.88	<0.001
**Morocco**	26	3.77 (1.07)	7.19 (1.18)	3.42	13.92	<0.001
**Bahrain**	24	5.25 (0.98)	8.63 (0.70)	3.38	15.11	<0.001
**Egypt**	65	3.72 (1.14)	6.97 (1.35)	3.25	20.89	<0.001
**Saudi Arabia**	44	4.24 (1.25)	7.43 (1.57)	3.19	15.45	<0.001
**Total Overall**	386	3.67 (1.13)	7.50 (1.25)	3.83	61.22	<0.001

Scores are based on a 10-point scale. SD = Standard Deviation. All *p*-values are < 0.001.

**Table 3:** Participant Satisfaction and Training Utility Evaluation (N = 386).

Evaluation Category	Assessment Criteria	Excellent (5)	Very Good (4)	Good (3)	Fair/Poor (1–2)	Mean
**Training Content**	Objectives clearly defined	100% (386)	0% (0)	0% (0)	0% (0)	5.00
	Relevance to clinical needs	95% (367)	5% (19)	0% (0)	0% (0)	4.95
	Time allotment	82% (316)	18% (70)	0% (0)	0% (0)	4.82
	Helpful materials	98% (378)	2% (8)	0% (0)	0% (0)	4.98
	Well-organized	100% (386)	0% (0)	0% (0)	0% (0)	5.00
**Trainer**	Questions answered effectively	100% (386)	0% (0)	0% (0)	0% (0)	5.00
	Presentation style	100% (386)	0% (0)	0% (0)	0% (0)	5.00
	Trainer knowledge	100% (386)	0% (0)	0% (0)	0% (0)	5.00
**Facilities**	On-site assistance/resources	75% (290)	25% (96)	0% (0)	0% (0)	4.75
	Quality of materials	96% (370)	4% (16)	0% (0)	0% (0)	4.96
**Overall**	Beneficial for growth	100% (386)	0% (0)	0% (0)	0% (0)	5.00

Values represent the percentage and frequency (n) of participants per rating. Ratings are based on a 5-point Likert scale where 5 = Excellent and 1 = Poor.

**Table 4:** Association Tests for Factors Influencing Knowledge Improvement (ANOVA).

Factor	Degrees of Freedom (df)	F-Statistic	*p*-Value	Effect Size (ηp^2^)
**Profession**	8	5.940	<0.001	0.11
**Employment Status**	1	6.473	0.0017	0.08
**Level of Education**	1	6.446	0.0115	0.06
**Previous AT Training**	1	4.958	0.0266	0.05

AT: Assistive Technology. Significance level set at *p* < 0.05.

**Table 5:** Thematic Analysis of Trainee Perceptions on Assistive Technology Barriers and Facilitators.

5 P Component	Key Barriers Identified	Representative Participant Feedback	Proposed Strategies
**Products**	Inadequate quality; supply chain issues	*“Limited access to high-quality products locally.”*	Local manufacturing; repair and refurbishment
**Provision**	Lack of accessible info; poor referral systems	*“The path from assessment to delivery is broken.”*	Integration across ministries; procurement mechanisms
**Personnel**	Lack of trained staff and time	*“We need more people who understand the fitting process.”*	Pre-service training; involving caregivers
**Policy**	Lack of user-involvement guidelines	*“Decisions are made for users, not with them.”*	National monitoring; consistent financing
**People**	Social stigma and discrimination	*“Patients are sometimes embarrassed to use devices in public.”*	Promoting social acceptance; advocacy for rights

## Data Availability

The data presented in this study are available on request from the corresponding author due to restrictions related to privacy and to ensure participant confidentiality.

## Supplementary Materials

The following supporting information can be downloaded at: https://www.mdpi.com/article/10.3390/healthcare14101362/s1.
