# Supplementary material for: The Effectiveness and Usefulness of Assistive Technology Training in Building Workforce Capacity for Rehabilitation and Healthcare Professionals in the MENA Region: A Mixed-Methods Study
Source: Healthcare (Basel). 2026 May 15;14(10):1362. doi: 10.3390/healthcare14101362 (PMC13205356; doi:10.3390/healthcare14101362)
Supplement: Supplementary file 1 [file healthcare-14-01362-s001.zip › healthcare-4279730-supplementary.pdf]

Trainee Name:

Assistive Technology Training Program (ATTP)

Pre-Post-Test (10 Questions, 15 Minutes)

1. **Parts (A and B):**

A. **When selecting an assistive product with a person, you should:**

- ○ Give the person any assistive product you have available
- ○ Find out about their health, lifestyle, which product they prefer, and where they will use the assistive product
- ○ Not involve the person in selecting the assistive product, it is best for you to select for them
- ○ Not involve the caregiver or family members of the person
- ○ Don't know

B. **The key steps involved in providing assistive products are:**

- ○ Select the assistive product and give it to the person
- ○ Select the assistive product that best meets the person's needs, fit the assistive product, teach the person how to use and look after the assistive product, and provide follow up
- ○ Tell the person what assistive product they need and where they can purchase it
- ○ Check what you have in stock and provide the person with any assistive product you have
- ○ Don't know

2. **Ahmad has a spinal cord injury and needs some help to move his body from his wheelchair into the car. What type of mobility assistive product could be useful?**

- ○ Walking aid
- ○ Portable ramp
- ○ Grab rail
- ○ Transfer board
- ○ Don't know

Trainee Name:

3. Which type of mobility assistive product could be useful to help someone to safely step in and out of the shower?

- Grab bar
- Transfer board
- Therapeutic footwear
- Wheelchair
- Don’t know

4. Sarah is 47 and has cerebral palsy. Ngosi walks with a walking frame and has two small steps on a landing to reach her bathroom.

The available space can fit ramp ratios 1:8, 1:10, and 1:12.

Which ramp ratio would be easier for Sarah to walk up?

- 1:8
- 1:10
- 1:12
- Don't know

5. A lady comes to you for a vision screen. Through the screening process you identify that she may have low vision, however she has never had a full eye examination by a health professional. What should you do?

- Provide her with a white cane
- Refer her to an eye health professional for a full eye assessment. Depending on the results, low vision assistive products may then be provided.
- Provide her with reading glasses
- Tell her that she does not require any assistive products

6. Maria has low vision and wears prescription glasses. She has difficulty seeing text unless it is very close, but would like to read books independently. She has arthritis and can't use her hands easily. Maria might benefit from a:

- ○ Handheld telescope
- ○ Stand magnifier
- ○ Handheld magnifier
- ○ White cane
- ○ Don't know

7. Toilet chairs are useful for people who:

- ○ Can control when they empty their bladder and bowel
- ○ Are incontinent and use a catheter
- ○ Find it difficult to get to or from the toilet
- ○ Find it hard to get on and off the toilet
- ○ All of these (except 'don't know')
- ○ Don't know

8. Parts (A and B):

A. The following items are all examples of assistive products that can help a person eat or drink:

- Modified cutlery
- Plate guards
- Cut away cups
- Cups with handles or spouts

- ○ True
- ○ False

B. The following items are all examples of assistive products that can help a person get dressed or undressed:

- Dressing stick
- Long handled shoe horn
- Sock aid
- Button hook and zip puller

- True
- False

9. Parts (A and B):

A. Which of these features is useful for people who need more support when showering?

- A removable bucket
- Castor wheels (wheels that turn / swivel) with brakes
- A backrest
- All of the above
- Don't know

B. If a person has any difficulty learning how to use their dressing aid, when should you follow up?

- 1-2 days
- 1-2 weeks
- 1-2 months
- 6 months

10. Parts (A and B):

A. What is the name of the dressing aid in the picture below?

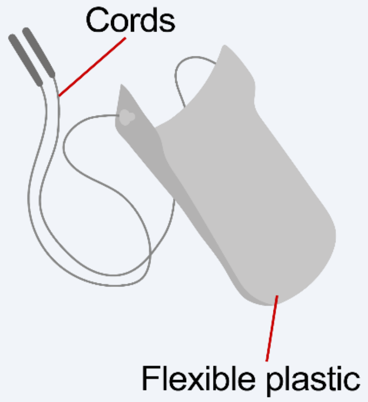

- Sock aid
- Dressing stick
- Button hook and zip puller
- Reach and grab tool

B. Rami is six years old and has cerebral palsy. He had to leave a shared home with his caregivers because of fighting. He cannot sit without support and is not able to walk. He has never had a wheelchair.

Does Rami need a wheelchair for long term or short-term use?

- Long term
- Short term
- Don't know

Assistive Technology Training Program (ATTP) Evaluation Survey

Please tick the box that represents how you would best rate the training program per the criteria mentioned below:

**Training Content**	Poor (1)	Fair (2)	Good (3)	Very Good (4)	Excellent (5)
Objectives of the training were clearly defined.					
Training was relevant to my needs & met my expectations.					
The time allotted for the training was sufficient.					
Material provided was helpful for further understanding of the topics.					
Content of the training was well organized.					
Participation and interaction were encouraged.					
This training experience will be useful in my work.					

**Trainer Evaluation**	Poor	Fair	Good	Very Good	Excellent
All members participated in the training effectively and their queries/ questions were answered.					
The trainer's style of presentation was impressive.					
The trainer was knowledgeable about the training topics.					
The trainer was well prepared.					

**Facilities Evaluation**	Poor	Fair	Good	Very Good	Excellent
The training was timely announced.					
Time management during the training was effective.					
Break was satisfactory.					
On-site assistance &/or resources were available.					
Training materials were beneficial.					

**Overall Impression**	Poor	Fair	Good	Very Good	Excellent
Motivating					
Beneficial					
Well-Planned					
Well-Conducted					

Please write strong points you appreciated in this training:

____

Please write your comments or suggestions for improvement, if any:

____

Thank you for your participation!
